# Supplementary material for: Are carbon emissions trading and green financial instruments synergistic? -Comprehensive quantitative research based on content analysis
Source: PLoS One. 2024 Mar 7;19(3):e0298601. doi: 10.1371/journal.pone.0298601 (PMC10919601; doi:10.1371/journal.pone.0298601)
Supplement: S1 Data — (ZIP) [file pone.0298601.s001.zip › Data/地级市Z_F工作报告.zip!/║╙─╧/║╫▒┌╩╨/└┤╫╘╣1⁄2╓┌║┼í╛┬φ┐╦╩2╛▌═°í┐_2013.docx]

各位代表：

现在，我代表市人民政府，向大会作政府工作报告，请予审议，并请各位政协委员提出意见。

一、2012年工作回顾

2012年，在省委、省政府和市委的正确领导下，全市上下紧紧围绕1123”总体部署，持续创优势，提升促发展，经济社会发展呈现良好态势，完成了市九届人大五次会议确定的目标任务。预计全市生产总值比上年增长10%，全社会固定资产投资增长22%，规模以上工业增加值增长13%，公共财政预算收入增长16.6%，社会消费品零售总额增长16%。尤为可喜的是，一些重点工作取得了重大进展：以煤化一体化项目列入国家规划、富士康项目开工、鹤淇电厂奠基、航盛电子工业园开园为标志，项目建设实现新突破；以鹤壁新区获批建设为标志，城市发展揭开新篇章；以永优“浚单20”荣获国家科技进步一等奖、我市被确定为全国整建制推进粮食高产创建试点市为标志，现代农业迈上新台阶；以鹤壁海关和出入境检验检疫局成功获批为标志，开放招商平台建设达到新水平；以国家循环经济示范市和国家森林城市创建规划通过国家专家评审为标志，生态建设取得新进展，鹤壁的影响力和美誉度进一步提升。这些重大进展，极大地鼓舞和振奋了全市人民加快发展的信心，有效带动了各项工作的深入推进、全面提升。

项目建设招商引资成效显著。总投资1422亿元的220个市重点项目完成投资280亿元，为年度投资计划的108%，其中120个新建重点项目全部开工，62个重点项目竣工投产。成功举办一系列招商活动，引进省外资金188亿元，目标完成进度居全省前列；新增融资125.6亿元、增加47.8亿元。产业集聚区和园区建成区面积新增8.4平方公里、达到54.7平方公里，现代煤化工等6个行业列入省重大建设基地或重点产业集群。

城乡面貌明显提升。城镇化率预计达到51.5%。鹤壁新区规划建设全面展开，淇滨区南部片区建设提升、淇水湾片区基础设施建设、淇河高村坝以下河道综合治理等扎实推进，商务中心区、特色商业区规划建设进展顺利，高铁鹤壁东站、市艺术中心等建成启用。老城区改造累计拆迁300多万平方米、开工500多万平方米，进入规划建设管理全面提升新阶段。两县县城改造和重点镇建设取得积极进展，大运河申遗鹤壁段整治工作基本完成。新型农村社区健康推进，建成12个、安置2万余人，在建44个、涉及33.2万人。

现代农业再创佳绩。高产创建再创5项全国纪录，高标准良田建设顺利推进，粮食总产实现“九连增”。农业产业化进程加快，13个农业产业化集群全部纳入省规划，新培育省级龙头企业3家、农民专业合作社113家。全国农业综合标准化示范市建设扎实推进，国家级现代玉米种子研发基地建设取得积极进展，河南省玉米良种培育工程技术中心、科技特派员工作站成立并发挥作用，农业科技支撑作用持续增强。

生态环境不断优化。创建国家循环经济示范市迈出坚实步伐，一批重点循环经济项目竣工投产。造林绿化24.2万亩，森林覆盖率、林木覆盖率分别达到26.8%和29.2%。节能减排工作取得新进展，预计万元生产总值能耗下降5.5%，河流出境断面水质持续达标，城市饮用水源地水质达标率保持100%，空气质量优良率91%，保持全省领先水平。

改革创新扎实推进。市级公立医院医疗资源整合和县级公立医院综合改革顺利推进，县级以下公立医疗机构全部实施了国家基本药物制度。数字鹤壁综合服务中心正式启用，38个职能部门373项行政服务事项实现集中办理，市政府门户网站进入全国50强。成立了市文化广电新闻出版局，组建了市豫剧牛派艺术研究院，国有文艺院团改革基本完成。城市执法管理体制改革取得新进展。鹤壁高新技术产业开发区获省批复，仕佳光分路器芯片项目成为我省首个列入国家863计划的电子信息类项目。

人民生活持续改善。民生支出占财政支出的78.5%，省、市重点民生工程顺利推进。预计城镇居民人均可支配收入、农民人均纯收入分别增长12%和14%。城镇新增就业3.9万人。学前教育三年行动计划稳步推进，职教园区加快建设，职业技术学院成为河南省骨干高职院校，我市被评为省职业教育攻坚工作先进市。城乡居民养老保险覆盖率、城镇基本医疗保险参保率、新农合参合率分别达到94%、92%和99.6%，医疗保险财政补助标准和低保标准进一步提高。建成保障性住房1.8万套。市人民医院主病房楼开工建设。市第九届运动会成功举办。农村道路建成204.7公里。解决了7.5万人饮水安全问题。水库三期移民搬迁安置工作积极推进。人口计生工作扎实开展。社会治安形势、信访形势和安全生产形势保持基本平稳。

民主法制和精神文明建设得到加强。自觉接受人大及其常委会依法监督，接受政协民主监督，支持政协参政议政，办理人大代表建议52件、政协提案253件，人大代表、政协委员对办理情况满意和基本满意。法制建设全面开展，“六五”普法工作深入推进。精神文明建设持续加强，公众道德水平和城乡文明程度进一步提升。突出抓落实这一主题，持续转变政府职能，扎实开展作风纪律整顿活动，行政效能和公务员素质进一步提高。深入推进政府廉政建设，反腐倡廉工作取得新成效。

同时，加强国防教育和国防后备力量建设，拥军优属、拥政爱民取得新成效，驻鹤解放军和武警部队在支持地方发展中作出了重要贡献。旅游、人防、外事、侨务、对台、民族宗教、统计、史志、档案等工作取得新成绩。对口援助哈密工作取得新进展。

各位代表！过去的一年，在严峻复杂的形势下，我们始终清醒忧患、满怀信心，始终凝心聚力、攻坚克难，始终务实重做、干事创业，经济社会发展取得新成效，人民生活水平得到新提高。这些成绩来之不易，是省委、省政府和市委正确领导的结果，是市人大和市政协关心支持的结果，更是全市人民团结奋进的结果。在此，我代表市人民政府，向工作在全市各条战线上的工人、农民、知识分子、干部、解放军指战员、武警官兵、公安民警以及各界人士致以崇高的敬意，向关心支持我市现代化建设的朋友们表示衷心的感谢！

各位代表！在肯定成绩的同时，我们也清醒认识到经济社会发展中还存在一些突出问题：经济总量小、竞争力较弱的基本市情尚未根本改变，产业结构不够优，质量效益不够高，资源型产业企业比重大，主要产业产品多处于产业链的中低端，服务业发展与全市发展和群众生活还不适应；城镇建设任务艰巨，老城区基础设施和公共服务设施欠账很多，城镇化质量有待提高；城镇居民收入较低，还有一定数量的贫困人口，就学、就医和社会保障等依然存在不足；政府自身建设还存在薄弱环节，一些干部的宗旨意识、服务能力、工作作风等与新形势新任务新要求还有差距。对此，我们一定增强忧患意识、责任意识，在今后工作中认真解决。

二、2013年工作总体要求

今年是全面贯彻落实十八大精神和实施《中原经济区规划》的开局之年，是实施“十二五”规划承上启下的关键一年，是为全面建成小康社会奠定坚实基础的重要一年。从当前形势看，一方面，国内外产业加速向我国中西部地区转移的态势没有改变，我市仍处于开放招商、承接产业转移的“黄金期”；国家和省里的工作总基调、政策措施和发展态势没有改变，这为我市加快发展提供了良好的环境；我市在中原经济区建设中的战略定位更加明确，干事创业、团结和谐、风清气正的氛围更加浓厚，这为先行先试走在前面提供了有力保证。另一方面，世界经济进入深度转型调整期，我国经济进入实质转型期和个位数增长阶段，区域竞争更加激烈，要素约束日趋加剧，这些都对我市发展带来不利影响。综合判断，我市正处于负重爬坡、不进则退、攻坚转型、务求突破的关键阶段，既面临难得的历史机遇和有利条件，也存在不容忽视的诸多困难和严峻挑战，我们的发展还任重道远。我们将坚定信心、抢抓机遇，努力赢得主动、赢得优势，在中原经济区建设中当先锋作示范。

今年政府工作的总体要求是：全面贯彻落实省委、省政府和市委的决策部署，以科学发展观为指导，以提高经济增长质量和效益为中心，围绕在中原经济区建设中先行先试走在前面，坚持人民至上、率先崛起，加快构建新型城市骨架和新型产业基础，在加快信息化进程中积极探索“两不三新”三化协调科学发展路子，持续创新，提升发展，实现经济健康较快发展和社会大局和谐稳定，迈出全面建成小康社会新步伐。

市委八届四次全会提出，到2020年在全省率先全面建成小康社会，主要经济指标增速高于全省平均水平、人口自然增长率低于全省平均水平，提前实现生产总值和城乡居民人均收入比2012年翻一番，人均生产总值和城乡居民收入力争赶上或超过全省、全国平均水平。根据这一要求，我们确定今年全市经济社会发展主要目标是：地区生产总值增长10%左右，全社会固定资产投资增长21%，规模以上工业增加值增长13%，引进省外资金200亿元，公共财政预算收入增长12%，城镇居民人均可支配收入、农民人均纯收入增长10%以上，社会消费品零售总额增长16%，城镇新增就业3万人以上，人口自然增长率控制在6.5‰以内，居民消费价格涨幅控制在3.5%以内，城镇化率提高1.8个百分点，节能减排完成省下达任务。

实现以上要求和目标，政府工作必须坚持在中原经济区建设中先行先试走在前面”的战略定位，努力在新型城镇化引领、产业转型升级、粮食生产和现代农业发展、文化发展繁荣、民生改善和促进社会和谐、生态建设和环境保护等方面，求创新、求突破、求先行。把握持续创新，提升发展”的总基调，就是把创新驱动放在全市发展的核心位置，进一步强化创新发展理念、提高创新发展能力、不断破解发展难题，加快建设创新型鹤壁，增强创新驱动发展新动力；就是牢牢把握发展第一要务，加快转变经济发展方式，促进产业转型升级，实现经济有规模、有质量、有效益、可持续的增长。把握“四个着力”的总要求：着力扩投资，坚持项目建设主抓手不动摇，坚持深化开放招商不止步，持续扩大民间投资和绿色投资，促进投资结构优化和质量效益提高，发挥好投资对经济增长的关键作用。着力促转型，把发展建立在创新驱动、集约高效、环境友好、内生增长的基础上，改造提升主导产业，培育壮大先导产业，加快发展服务业和现代农业，持续做特色、创优势、出精品。着力抓改革，把改革创新作为加快发展的关键，积极推进体制机制改革，落实好已出台的改革措施，最大限度地调动方方面面的积极性，最大限度地激发各类市场主体的创造力和内在活力，加快培育形成发展的新动力。着力惠民生，把人民放在心中最高位置，按照“守住底线、突出重点、完善制度、引导舆论”的基本要求，更加扎实地推进涉及民生的各项工作，让发展成果更多更公平地惠及全市人民。

三、2013年重点工作

做好今年的工作，我们将立足全局、统筹兼顾，以创新工作理念、思路、机制、方式方法推动工作、提升发展。着力做好以下几项工作：

一是突出项目建设和招商引资。持续做大做强存量、招大引优增量，以项目建设和招商引资带动全局工作的提升。强力推进项目建设。集中力量抓好总投资1530亿元的260个重点项目，完成投资340亿元，特别是抓好鹤淇电厂等37个超10亿元的重大项目，力争煤化一体化等160个新建重点项目开工、中鹤20万吨速冻调理食品等50个项目竣工。谋划储备一批举全市之力来推进的大项目好项目，力争新谋划超10亿元项目50个以上。持续提升招商实效。围绕发展主导先导产业，突出重点招商项目，着力引进央企、行业龙头、知名品牌和延链补链项目，力争在招大商、引大资上取得更大突破。突出重点招商区域，加强中原经济区内合作开放，抓住高铁经济圈、郑州航空港经济综合实验区建设等机遇，主动融入，借势发展。在扩大第二产业招商规模和质量的同时，加大一产、三产方面的工作力度，强化对落地项目的后续服务，推动开放招商向更宽领域、更高水平迈进。打造完善载体平台。把培育产业集群作为提升产业集聚区发展水平的主攻方向，突出龙头带动、链式发展，培育形成多个超百亿元产业集群。坚持“筑巢创优”，围绕企业发展需要，加快完善产业集聚区和园区基础设施公共服务设施，积极推进产业集聚区创建国家级、省级新型工业化示范基地等。持续提升配套能力，加快开放平台建设，力争海关、出入境检验检疫局建成投用，积极推进金属镁、食品等出口基地建设。

二是加快产业转型升级。坚持做大总量和优化结构并重，注重从量的积累转向质的提升，加快构建新型产业体系。做优做强主导先导产业。坚持集群集聚、拉长链条、完善配套、打造品牌，煤电化材一体化产业强化市、区协同发展，重点推动实施煤制烯烃、煤制乙二醇和甲醇、电石等一批下游产业项目，形成建设发展高潮。食品加工产业加快实施速冻调理、快餐休闲食品等大项目。汽车及零部件产业着力推进整车、改装车、关键零部件规模化总成化发展。电子信息产业重点发展汽车电子、光电子、军工电子和智能终端消费电子，努力打造国家级电子新城。金属镁精深加工产业加快发展高品质镁合金和镁合金终端产品，推进国家镁及镁合金产品质量监督检验中心资格认证工作，建设河南省轻金属高科技中心，打造世界一流的金属镁精深加工基地。大力发展服务业。把服务业发展作为加快两个构建的应有之义和重要支撑，着力提升层次、强化配套、促进集聚、打造品牌，带动服务业发展提速、水平提升、比重提高，更好地服务企业生产和群众生活。大力发展现代物流等生产性服务业，抓好物流项目和园区建设，增强物流集散功能；积极发展金融保险、规划设计、咨询评估等现代服务业。加快发展商贸、旅游、养老康复等生活性服务业，抓好商务中心区、特色商业区等服务业集聚区建设，推进新区商圈规划建设和传统商贸业升级改造；有效融入中原经济区和更大范围的旅游格局，谋划建设南太行5A级景区，引进和发挥好龙头旅行社的带动作用，完善旅游产业链条和配套服务，提升旅游业发展层次。加快提高信息化水平。抓住中原经济区打造中部地区新一代信息技术产业基地的机遇，加快建设新型信息基础设施，提升信息技术管理应用水平。深化信息化与工业化融合，加大“两化”融合示范企业培育力度，积极发展信息服务业和信息制造业。深化信息化与城镇化融合，积极创建国家智慧城市试点，进一步整合政务服务平台及政务信息化资源，深化空间地理框架信息系统应用，建成数字城管系统，实现3G网络城乡全覆盖。深化信息化与农业现代化融合，抓好数字农业综合服务平台建设，加快中国鹤壁农业硅谷产业园建设，积极打造农业高科技示范基地和产业集聚区。

三是加快新型城镇化进程。把扩大规模与提高质量、加快建设与集聚人口更好地结合起来，高标准推进鹤壁新区规划建设，力争为“三年初见成效，五年大见成效”开好局、起好步；进一步发挥城市核心区的龙头带动作用、老城区和浚县县城组团的助推协同作用、重点镇和新型农村社区的有效支撑作用，推进城乡规划、基础设施建设、公共服务等一体化，实现高效率、高品质、高水平的发展。加快新型城镇体系建设。统筹推进新城建设与建成区功能完善、品位提升，鹤壁新区按照“三区一基地”定位，坚持南北相向发展、中间产业连接，加快推进规划编制、基础设施和淇水湾片区等重点项目，全面掀起建设高潮；加快淇滨区南部片区、商务中心区、高铁片区等规划建设步伐，有序推进淇滨区建成区、淇县县城和城中村改造建设。强化整体规划和组织协调，更高层次统筹推进老城区建设发展，力争在产业发展、城市建设、公共服务、生态文明等方面取得实质性进展；加快浚县黎阳新城建设，结合大运河申遗，抓好古城保护开发。扎实推进重点镇基础设施建设和产业发展，提升综合承载能力和经济实力。积极稳妥推进续建的44个新型农村社区，力争10个社区部分达到入住条件；加快集聚区内村庄搬迁改造。提升城市功能品位。加强综合交通体系与城镇化布局的有机衔接，服务好晋豫鲁铁路建设，加快建设鹤辉高速，完成107国道升级改造、永定线改建等工程，开工大海线、浚南线改建等浚县与中心城区间快速通道项目，加快推进高铁汽车客运枢纽站等交通场站建设，提高农村公路质量；增加城乡公共交通站点和车次，提高服务质量和水平。强化精品理念和服务管理，提升亮化、绿化、净化水平和水电气热综合保障能力，提升城市服务管理政策措施的针对性、操作性和执行力，提升社区物业服务管理的精细化和个性化水平，打造整洁有序、宜居舒心的环境。保护利用好具有历史文化价值的建筑设施，传承延续城市文脉。创新工农城乡利益协调、财产确权、户籍、就学、社会保障等制度，保护农民合法权益，有序推进农业转移人口市民化。完善城乡规划体系。加强城乡规划与产业布局、土地利用规划、综合交通运输规划相互衔接，完成鹤壁新区总体规划和商务中心区、特色商业区空间规划及控制性详规，完善水系、停车场、环卫等专项规划，提高规划的人本性、超前性和系统性。

四是加快现代农业发展。坚持主攻单产、改善品质、提高效益，促进粮食增产、农业增效、农民增收。深入推进整建制高产创建。加快小麦、玉米万亩示范片建设，力争高产创建面积达到82万亩，有序推进高产创建田向高产良种田转变。有效实施国家新增千亿斤粮食生产能力规划工程、中低产田改造等，建设高标准粮田16万亩。积极推进火龙岗地区高效节水灌溉示范基地、小型农田水利等农业基础设施建设。强化农业科技支撑能力。以国家农业科技园区申报建设为抓手，着力推进种子研发中心、国家级农业气象试验站、农业物联网信息技术研发装备中心、国家区域新品种展示中心基地建设，加快推进卫星遥感应用等气象专项，做大做强“永优”种业品牌，打造现代农业科技新亮点。提升畜牧养殖科技水平，争创一批国家、省级标准化规模养殖示范场。以创建全国农业综合标准化示范市为载体，大力提升农业标准化水平，确保农产品安全。提高农业产业化水平。继续实施农业产业化集群培育工程，发展创意、休闲等新型农业，积极申报省优质农产品示范基地。探索新型农业经营体系，培育新的经营主体，发展多种形式规模经营，引导土地规范有序流转，鼓励发挥好种养大户、家庭农场、农业产业化龙头企业和农民专业合作社的作用。

五是加快文化发展繁荣。充分发挥我市文化资源比较优势，大力推进文化事业、文化产业发展，提高城市的文化内涵和品位，激发广大人民群众生活热情和创新活力。深化文明创建工作。积极开展社会主义核心价值观宣传教育活动，弘扬鹤壁精神，推进社会公德、职业道德、家庭美德、个人品德建设，加强青少年思想道德建设。深化群众性精神文明创建工作，高标准制定文明创建目标任务，积极开展志愿服务、季评十件好人好事等活动，树新风、育新人，提高全民文明素质和社会文明程度。积极发展文化事业。完善覆盖市县乡村的公共文化服务体系，继续实施文化惠民工程，发挥好乡镇文化站、农村文化大院、农家书屋、社区文化活动室等公共文化设施的作用，扎实做好舞台艺术进基层活动和农村电影放映工程，广泛开展城乡群众性文化活动，为人民提供更好更多的精神食粮。大力发展文化产业。突出浚县古城保护开发，叫响国家历史文化名城品牌；突出中国鹤壁民俗文化节，打造独具特色的中原民俗经典盛会。挖掘和宣传推介特色文化资源，打造文化精品和特色体育品牌。加快文化产业园区建设，谋划引进一批文化产业重大项目和重点企业，提高文化产业规模化集约化专业化水平。

六是加快生态文明建设。把生态文明理念融入经济社会发展各方面和全过程，扩大绿色生态空间，推进绿色发展、循环发展、低碳发展，积极申报建设国家可持续发展实验区，打造生态宜居的美丽鹤壁。全面提升循环经济发展水平。深入推进国家循环经济示范市、国家资源综合利用示范基地等创建工作，力争列入首批国家循环经济示范市；按照纵向衔接、横向耦合、区域循环、低碳生态的发展理念，加快产业集聚区、循环经济项目、试点企业和示范园区建设，提高城市中水循环利用水平，完善循环型工业、循环型农业、循环型城市三位一体的循环经济体系；提升循环经济标准化水平，争取更多的标准上升为国家或行业标准，继续走在前列、作出示范。扎实推进节能减排。进一步降低能源、水等消耗强度，提高利用效率和效益。严控新上高耗能项目，抓好企业节能，坚决淘汰落后产能；继续推进既有居住建筑节能改造，启动国家节能减排综合示范市创建工作；支持节能低碳产业和新能源、可再生能源发展；抓好工业污染防控和养殖企业污染治理，强化城镇污水、垃圾处理设施建设和运行管理，确保淇河不受到污染，城市饮用水源地水质达标率保持100%，空气质量优良天数保持全省领先水平。持续加强生态建设。积极创建国家森林城市、国家绿色生态城市、中美低碳生态试点城市，加快推进覆盖全域的林业生态体系建设，全年造林绿化47万亩，森林覆盖率达到31.5%；加快以淇河为龙头的生态城市建设，增强生态产品生产能力。加快沿淇河风貌带和绿色生态走廊建设，积极申报盘石头水库湖泊生态环境保护试点，打造富有特色的生态休憩地。加强农村环境连片治理，解决好农村居民饮水安全问题。推进土地综合整治和矿山地质环境治理。积极营造生态文明氛围。落实完善资源节约、生态环境保护、生态文明建设的政策措施，严格环境监管，解决好关系群众切身利益的环境问题，加强生态文明宣传教育，增强全民节约、环保和生态意识。

七是深入推进改革创新。进一步激发释放“改革红利”，加快推进体制机制、科技、要素保障、社会管理等创新，增强发展的动力活力。扎实推进重点领域改革。深化行政审批制度改革，清理整顿和规范涉企行政事业性收费。深化医药卫生体制改革，加快推进全民医保，完善基层公共卫生服务体系，巩固基本药物制度实施效果，深入推进县级公立医院综合改革。加快文化体制改革，推进经营性文化事业单位改制，引导更多民间资本进入文化领域。积极推进财税体制、收入分配、资源产品价格、农电体制等改革。加大科技创新力度。充分发挥企业创新主体作用，组织实施好国家和省重大科技专项，加快建设科技孵化器、关键技术研发中心等创新平台，深化与高等院校、科研院所的合作；促进创新资源高效配置和综合集成，增强科技对经济发展的核心支撑能力。加快全国质量强市示范市创建工作，推进质量提升、标准化和品牌建设。创新要素保障机制。提高建设用地保障能力，探索开展农村土地综合整治、城乡建设用地增减挂钩和人地挂钩试点工作；强化依法依规和节约集约用地，加大闲置、低效利用土地及资产盘活利用力度；加快产业集聚区标准厂房、集合式办公设施等建设。完善银政企合作长效机制，探索开展农村金融综合改革创新试点工作，积极推进农商行组建、村镇银行建设和域外股份制银行引进工作；推动鹤壁银行改革发展，争取实现跨区经营；扩大社会融资规模，引导规范民间资本发展，加大企业上市培育和帮扶力度；依法加强金融监管，持续营造良好金融环境。大力发展民营经济。把民营经济发展放在重要战略位置，与项目建设、招商引资、产业发展、县域经济等更好地统筹协调，落实完善政策措施，持续改进服务、优化环境，发挥行业协会、商会等作用，大力支持小微企业发展，促进民营企业不断扩规模、上水平。持续优化企业服务。健全企业服务长效机制，定期开展逐县区巡回现场办公活动，帮助企业解决实际困难和问题，促进企业持续做大做优做强。

八是着力保障和改善民生。从解决人民群众的长远利益、根本利益着眼，从人民群众的现实利益、具体利益着手，抓好省市重点民生工程，让发展成果更多更公平惠及人民群众。多渠道促进居民增收。落实好强农惠农富农政策，加大扶贫开发尤其是西部山区贫困人口脱贫力度，努力缩小城乡居民收入差距；健全职工工资正常增长机制和支付保障机制，落实城镇最低工资标准，完善企业工资集体协商制度，努力实现城乡居民人均收入增长与经济增长同步。推进实现更高质量的就业。全面落实促进就业政策，支持自主创业，健全就业服务网络，做好以高校毕业生为重点的青年就业工作和农村转移劳动力、城镇困难人员、退役军人就业工作，动态消除“零就业家庭”，促进更多劳动力就地就近就业。深入实施人才引进计划，引导企业健全用人长效机制，创造更好的吸引人才、使用人才、留住人才的环境，吸引更多人员到我市创业就业。保护劳动者合法权益，构建和谐劳动关系。努力办好人民满意的教育。扎实推进学前教育，全面实施学前教育三年行动计划。促进基础教育均衡发展，继续推进优质教育资源整合扩容，规划兴建中小学生综合实践基地，改善农村尤其是山区义务教育薄弱学校办学条件，解决好校园安全、“大班额”等问题。继续实施职业教育攻坚，加强职教园区、职业技术学院等基础能力和内涵建设，引导职业院校结合产业发展调整优化专业设置，做好鹤壁能源化工职业学院高职申报工作。支持社会力量办学，推进民办教育发展。增强社会保障能力。扩大社会保险覆盖面，确保养老金、失业金等社会保险待遇按时足额发放。健全城乡社会救助体系，提高城乡低保、农村五保供养标准和优抚对象待遇水平。加大现有乡镇敬老院改造，探索实行集中供养全覆盖试点，提高养老服务水平。完善住房建设发展规划，加强保障性住房建设和管理，新开工2.1万套、建成1.3万套，促进房地产健康发展。完成盘石头水库三期移民搬迁安置工作。积极发展社会福利、慈善、老龄、妇幼和残疾人事业，做好流浪人员救助工作。加快殡仪馆、公益性公墓等规划建设。提高医疗卫生水平。加快市人民医院主病房楼、市中医院病房楼等建设，启动市人民医院申报三甲医院工作，持续提高医疗服务质量；新农合和城镇居民基本医疗保险财政补助标准由每人每年240元提高到280元，人均基本公共卫生服务标准由25元提高到30元；完善基本药物制度，确保全市县级以下医疗服务机构实施基本药物制度全覆盖；大力支持社会资本举办医疗机构；启动国家卫生城市创建工作。深入实施全民健身计划，增加群众体育活动场所。稳定低生育水平，提高出生人口素质，促进人口长期均衡发展和人的全面发展。保持社会和谐稳定。加强和创新社会管理，坚持依法办事和依法维权相结合，畅通群众诉求渠道，改进服务方式方法，及时解决群众反映的热点、难点问题，提高社会服务管理水平。深入推进平安建设，高度重视安全生产和食品药品安全，提高应急处置能力，提升人民群众安全感和满意度。

持续加强国防教育和国防后备力量建设，增加驻鹤解放军、武警部队、民兵预备役和人民防空基础能力建设投入，深入开展双拥共建，促进军民融合式发展。支持工会、共青团、妇联等人民团体发挥桥梁纽带作用，注重发挥工商联等社团作用。做好外事、侨务、对台、民族宗教、统计、史志、档案、援疆等工作。

四、加强政府自身建设

形势在变化，发展在转型，社会在调整，要求政府进一步提升科学发展能力、运作执行能力，要求政府工作人员进一步提升自身素质、改进工作作风，努力建设为民务实清廉的服务型政府。

一是坚持人民至上。增强宗旨意识，认真倾听群众呼声，真情关心群众疾苦，凡是涉及群众切身利益的决策都要充分听取群众意见，凡是损害群众利益的做法都要坚决防止和纠正。多办实事好事，多深入基层和一线，多到困难和矛盾突出的地方去，解决群众生产生活中的实际问题。提升服务水平，为人民群众和市场主体提供更加优质的服务，让群众得到更多实惠，让社会更加公平正义。

二是严格依法行政。加快转变政府职能，深入推进政企、政资、政事、政社分开，注重发挥市场、企业、社会组织和中介组织的作用。敬畏和用好人民赋予的权力，依法依规履职尽责，做好“六五”法制宣传教育和依法治市工作，提高规范公正文明执法水平，建设法治政府。认真执行人大及其常委会的决议、决定，自觉接受人大依法监督、政协民主监督和群众监督、舆论监督，认真办理人大代表议案、建议和政协提案，广泛听取各民主党派、工商联和无党派人士意见建议。完善政务公开和办事公开，让权力在阳光下运行，提高政府的执行力和公信力。

三是切实改进作风。全面落实改进工作作风、密切联系群众的各项规定，扎实开展为民务实清廉教育实践活动，着力解决慵懒散奢等作风问题。坚持干实事、求实效，说到做到、说好做好，以务实重做推动实实在在的发展。坚持提高效率、担当有为，做到事不避难、事不避险、事不避重，不断拉高工作标杆，争创一流业绩。坚持艰苦奋斗、勤俭节约，牢固树立过紧日子的思想，严格控制行政经费等一般性支出，切实降低行政成本，确保每一分资金都用在促进发展、造福群众上。

四是加强廉政建设。落实完善制度，提高制度执行力，真正做到用制度管权、管事、管人。严格实施追究，对政府系统工作人员严格教育、严格监督、严格管理，强化行政监察、审计监督等工作力度，严肃查处慢作为、不作为、乱作为。营造良好氛围，认真落实廉政建设责任制和廉洁自律各项规定，自觉接受组织、社会、人民群众等方方面面监督，保持干事创业、团结和谐、风清气正的好环境。

各位代表！我市发展已经站在一个新的历史起点上。面对经济社会发展的新形势新任务，面对广大人民群众过上美好生活的新期盼新要求，我们深感责任重大、使命光荣。让我们在省委、省政府和市委的正确领导下，进一步解放思想、开拓创新，攻坚克难、扎实工作，全面完成今年各项目标任务，为率先全面建成小康社会而努力奋斗，为加快中原崛起河南振兴作出应有贡献！
